# Good vibrations: Exploring how orbital resonance shapes the Galilean moons

**DOI:** 10.1038/s41467-026-72078-4

**Published:** 2026-05-11

**Authors:** Allard Veenstra, Quirijn van Woerkom, Alessandra Marzolini, Sam Fayolle, Valerio Filice

**Affiliations:** 1 Faculty of Aerospace Engineering, TU Delft, Delft, HS The Netherlands; 2 European Space Agency (ESA), European Space Research and Technology Centre (ESTEC), Noordwijk, AZ The Netherlands

**Keywords:** Rings and moons, Geodynamics

## Abstract

Upcoming missions to the Galilean moons will revolutionize our understanding of their interior evolution, largely governed by their orbital resonance. Yet, to fully understand the feedback between orbit and interior, crucial in determining their habitability potential, continued efforts are needed.

## From discovery to first characterization

**Fig. 1:**
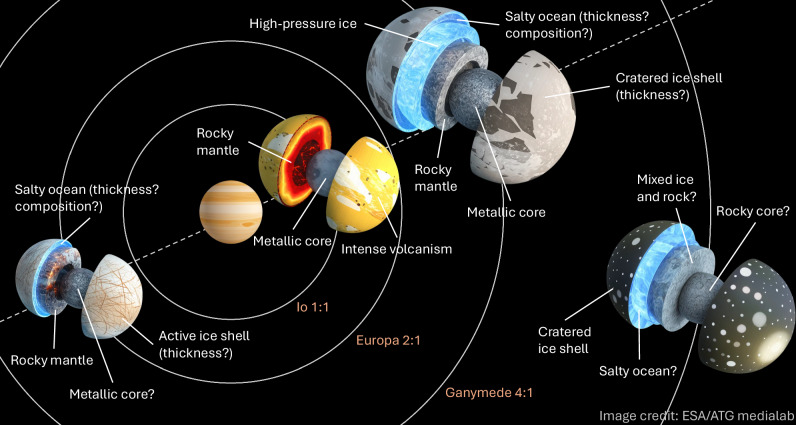
Schematic illustration of Jupiter and the four Galilean moons, from the planet outwards: Io, Europa, Ganymede, and Callisto. Jupiter is not to scale, while the moons are scaled relative to each other. Current knowledge suggests distinct internal architectures for the Galilean moons: Io is characterized by a rocky mantle over a largely iron core, while Europa and Ganymede likely feature liquid water oceans above rocky mantles and solid cores. Callisto is thought to be only partially differentiated, with a putative ocean overlying a mixed interior of ice and rock. The Laplace resonance is indicated by the ratios and means that for every one revolution of Ganymede around Jupiter, Europa does two, and Io four. Illustrations adapted from material produced by ESA/ATG medialab, published in “Inside the Galilean Moons”, 2021.

Since Galileo Galilei first resolved four faint points of light orbiting Jupiter in 1610, the Galilean moons have served as a natural laboratory for understanding how planetary systems work. With increasingly powerful ground-based observations, it became clear that Io and Europa are roughly the size of Earth’s Moon, whereas Ganymede—the largest moon in the Solar System—is larger than Mercury, and Callisto’s size is in between; see also Fig. 1. Before that, long-term observations already showed that the inner three moons, Io, Europa, and Ganymede, are locked in a 4:2:1 orbital resonance (the so-called Laplace resonance), which forces their orbits to remain slightly eccentric (i.e., non-circular).

When the Voyager 1 and 2 spacecraft visited Jupiter in 1979, the consequences of this eccentricity became apparent: the three inner moons exhibited intense activity. Io showed widespread volcanism so extreme that its internal power output exceeds that of Earth^1^. Europa’s icy surface was nearly devoid of impact craters, implying a young and continually renewed surface, while its fractured terrain hinted at a liquid ocean beneath the ice shell. Ganymede also displayed complex tectonic structures, hinting at a strong reheating event in the distant past that overturned large parts of its surface^2^. In contrast, Callisto’s heavily cratered surface indicated a great surface age and limited internal activity^2^.

To investigate the system in greater detail, the Galileo spacecraft orbited Jupiter from 1995 to 2003. The estimate of the moons’ moments of inertia (MoI), based on the measured degree-two gravity field, revealed a significant difference in internal mass distribution between the Galilean moons, and hence indicating a gradient in internal differentiation: the interiors of Io, Europa, and Ganymede are divided into distinct layers with different chemical compositions and densities, whereas Callisto shows a lower density contrast between layers^3^. Magnetic induction measurements provided evidence for subsurface oceans beneath Europa’s ice shell and, surprisingly, also in the interior of Callisto^4^. For Ganymede, the Galileo mission revealed that this moon generates its own magnetic field in a (partially) liquid metallic core^3^, similar to Mercury and the Earth, which hides the induction signal partly^5^. The presence of a subsurface ocean inside Ganymede was later confirmed from aurora fluctuations observed by the Hubble Space Telescope^6^. The discovery of liquid water beyond the snowline fundamentally reshaped our understanding of planetary habitability and expanded the range of potentially habitable environments.

The evolution of the inner three Galilean satellites and their subsurface oceans is governed by the strong tidal interactions between Jupiter (precisely characterized by the Juno mission) and its moons. The long-lived Laplace resonance, linking Io, Europa, and Ganymede, maintains a strong feedback between orbital dynamics and interior evolution. As Jupiter’s gravity repeatedly stretches and compresses the moons along their eccentric orbits, internal friction generates heat, sustaining significant dissipation in Io and Europa over geological timescales. Differences in the magnitude and distribution of this tidal heating could explain the striking geological contrasts observed between the moons. Tidal heating also plays a fundamental role in the formation and long-term survival of their subsurface oceans, especially Europa’s. The source of this heating is the moons’ orbits, which are slowly evolving as a result^7^. Consequently, an understanding of the internal structures and evolution of these satellites is inseparable from the history of the Laplace resonance itself. Although there is a strong indication that Io has been tidally heated since formation^8^, the origin of the resonance remains debated. Whether the resonance is primordial or the result of a later capture implies markedly different thermal histories of the moons’ hydrospheres^9,10^. Characterizing the moons’ habitability potential today thus tells us little about their longevity if we cannot reconstruct the orbital-thermal feedback loops that led to them.

Each moon’s surface, interior, and dynamics encode signatures of different episodes of the resonance’s history. The first step in characterizing these is to determine the density, thickness, and composition of the moons’ internal layers (Fig. 1). Their internal differentiation is related to past and current heating, because establishing a density stratification requires high temperatures. Furthermore, the moons’ tidal response (i.e., their deformation under Jupiter’s gravity) is determined by their internal properties. The upcoming decade of exploration of the Galilean moons will provide invaluable constraints on the present state of the moons’ orbits and interiors.

## The 2030s: the era of detailed characterization

In the coming decade, three spacecraft will investigate Jupiter’s icy moons, Europa, Ganymede, and Callisto. ESA’s Jupiter Icy Moons Explorer (Juice), which will arrive in 2031, will have two close flybys of Europa, 20 of Callisto, and will ultimately orbit Ganymede^11^. NASA’s Europa Clipper will focus on Europa’s habitability and, starting in 2030, will conduct at least 49 close flybys of Europa, 7 flybys of Ganymede, and 9 of Callisto^12^. China’s proposed Tianwen-4 space mission launches around 2030 and will orbit Callisto^13^. Unfortunately, due to the harsh radiation environment, none of the three missions will come close enough to Io to do in situ and high-resolution observations. For broader overviews of the missions, see refs. ^11–13^.

The Europa Clipper mission^12^ is designed to assess Europa’s habitability. As part of this goal, it will quantify the ice shell’s thickness and the salinity and depth of the ocean through multi-frequency electromagnetic induction and ice-penetrating radar sounding, and Europa’s tidal response using both gravimetric and altimetric measurements. Combining these measurements will characterize Europa’s ocean and constrain the moon’s density distribution^14^. Europa Clipper will also resolve the properties of the outer hydrosphere and potentially detect seafloor gravity anomalies. In conjunction with interior characterization, the mission aims to detect and interpret prebiotic chemistry signatures. It will analyze Europa’s surface, specifically the chaos terrain, its atmosphere, and potential plumes through a combination of remote sensing spectroscopy and mass spectrometry of gases and dust in the exosphere^12^.

Juice will be the first mission to orbit a moon other than ours, which offers a unique opportunity to investigate the interior of Ganymede. Using a combination of the radio-science experiment and laser altimeter, Juice will measure Ganymede’s gravity field, topography, and librations, and determine its tidal response. Additionally, Juice’s magnetometer will separate Ganymede’s intrinsic magnetic field, most likely generated in its metallic core, from the magnetic signals induced in the salty ocean. Combining these measurements will accurately determine the properties of the moon’s hydrosphere and mantle, including the ice shell thickness and the depth and composition of the ocean^11^.

During the Callisto flybys, Juice will measure the gravity field from radio tracking, giving estimates of both its MoI and tidal response. Furthermore, Juice will be able to confirm the putative detection of a subsurface ocean^11^. The flybys of Juice and Europa Clipper will likely not be enough to determine the thickness of Callisto’s ice shell. Tianwen-4, however, because of its proposed low orbit of 200–400 km, will be able to measure it^13^.

Finally, the combined radio-science constraints from Juice and Europa Clipper will allow precise determination of any change in the moons’ orbits, thus providing estimates of the dissipation within Jupiter. Such an estimate will be crucial to determine the present orbital migration rate of each moon and reconstruct the recent past’s evolution of the Laplace resonance. For Callisto, this will unambiguously confirm or rule out a potential faster migration caused by the so-called “resonance-locking” mechanism^15^, where the dissipation in the host planet is enhanced at discrete frequencies dependent on the planet’s interior structure, linking satellites’ orbital evolution and planetary interior evolution [e.g., ref. ^16^].

The upcoming missions’ unprecedented measurement precision will enable a shift in research focus from standard one-dimensional radial models to more complex three-dimensional characterizations of planetary interiors [e.g., ref. ^17^]. To interpret these observations, self-consistent, coupled models that integrate thermal evolution with multiscale orbital dynamics need to be developed. These models will help reconstruct the history of tidal dissipation and internal stresses inside the moons. Through this analysis, we will finally have the opportunity to assess the longevity of the moons’ habitability potential.

## Beyond the next 15 years

After Juice, Europa Clipper, and Tianwen-4, we will have unprecedented characterization of Jupiter’s icy moons. Ganymede, due to Juice’s low orbit, will be the best-studied moon beyond our own. However, some fundamental aspects of the system will remain unknown. Specifically, the connection between Io and the other moons will not be studied in detail. Io, the main driver of the tidal evolution of the system via the Laplace resonance, also influences the surface chemistry of the other moons due to the large amount of material ejected from its atmosphere. These gaps are crucial for understanding habitability and interior and orbital evolution, as coupling between the orbits and interiors of Io and Europa can drive major changes in tidal heating^18^. Thus, to better understand the system’s dynamics, a dedicated mission to Io is crucial^19^.

Furthermore, future exploration must transition from orbital reconnaissance to surface-based strategies, such as sampling, seismometry, and deformation measurements. Therefore, the next generation of Jupiter missions must prioritize a lander on Europa to unambiguously resolve its deep interior structure^20^. Simultaneously, modeling efforts should be made to better understand the coupling between the interior and orbit.

The Jovian system offers more than studies of icy moons; the system is an analog for worlds that are too distant and faint to be studied directly. The icy Galilean moons are considered the best Solar System analogs for ocean worlds^21^, while Io provides an analog for hot close-in exoplanets, and serves as a present-day laboratory to study magma transport mechanisms that may dominate on young or large exoplanets^22^. Additionally, the resonant, compact orbital structure found in Jupiter’s moons appears common in exoplanetary systems around low-mass stars, pointing to a common formation pathway. The most notable example of such a system is TRAPPIST-1^23^, which hosts seven Earth-sized planets on orbits resembling those of the Galilean moons. Future study of the Galilean moons is therefore also important for comparative (exo)planetology.

There is still much to be gained from studying the Jupiter system. As stated beautifully by Smith et al.^2^ following Voyager 1’s visit (before the detection of exoplanets): “The sense of novelty would probably not have been greater had we explored a different solar system”.
